# Estimating occupancy of Chinese pangolin (*Manis pentadactyla*) in a protected and non‐protected area of Nepal

**DOI:** 10.1002/ece3.6198

**Published:** 2020-03-17

**Authors:** Sandhya Sharma, Hari P. Sharma, Chanda Chaulagain, Hem B. Katuwal, Jerrold L. Belant

**Affiliations:** ^1^ Himali Conservation Forum Taplejung Nepal; ^2^ Central Department of Zoology Institute of Science and Technology Tribhuvan University Kathmandu Nepal; ^3^ Pashupati Multiple Campus Tribhuvan University Kathmandu Nepal; ^4^ Center for Integrative Conservation Xishuangbanna Tropical Botanical Garden Chinese Academy of Sciences Mengla Yunnan China; ^5^ University of Chinese Academy of Sciences Beijing China; ^6^ Camp Fire Program in Wildlife Conservation State University of New York College of Environmental Science and Forestry Syracuse NY USA

**Keywords:** Chinese pangolin, farmland, food, habitat suitability, occupancy, red soil

## Abstract

Chinese pangolin is the world's most heavily trafficked small mammal for luxury food and traditional medicine. Although their populations are declining worldwide, it is difficult to monitor their population status because of its rarity and nocturnal behavior. We used site occupancy (presence/absence) sampling of pangolin sign (i.e., active burrows) in a protected (Gaurishankar Conservation Area) and non‐protected area (Ramechhap District) of central Nepal with multiple environmental covariates to understand factors that may influence occupancy of Chinese pangolin. The average Chinese pangolin occupancy and detection probabilities were $\hat{\Psi }$ ± *SE* = 0.77 ± 0.08; $\hat{p}$ ± *SE* = 0.27 ± 0.05, respectively. The detection probabilities of Chinese pangolin were higher in PA ( $\hat{p}$ ± *SE* = 0.33 ± 0.03) than compared to non‐PA ( $\hat{p}$ ± *SE* = 0.25 ± 0.04). The most important covariates for Chinese pangolin detectability were red soil (97%), food source (97.6%), distance to road (97.9%), and protected area (97%) and with respect to occupancy was elevation (97.9%). We recommended use of remote cameras and potentially GPS collar surveys to further investigate habitat use and site occupancy at regular intervals to provide more reliable conservation assessments.

## Graphical abstract

Chinese Pangolin detection probabilities is higher in protected areas than non‐protected area.

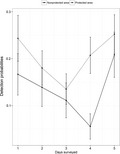

## 1 INTRODUCTION

The trend in global mammal population declines has highlighted the need for rigorous and extensive monitoring programs to document species occurrence and evaluate population change (WWF, 2014) for their long‐term conservation (Janssen & Leupen, 2019; Siddig, 2019). Governments and nongovernmental organizations are actively involved with long‐term, large‐scale studies to monitor flagship mammalian populations (e.g., Bengal tiger *Panthera tigris tigris*, One‐horned rhinoceros *Rhinoceros unicornis)* (Barber‐Meyer et al., 2012; DNPWC, 2009; Subedi et al., 2014) and develop conservation action plan (e.g., Tiger Conservation Action Plan (DNPWC, MoFSC, & GoN, 2007), Blackbuck Conservation Action Plan (DNPWC, 2016), and Pangolin Conservation Action Plan (DNPWC & DoF, 2018).

Among mammalian species, pangolins are globally threatened from population declines due to illicit trade (Challender, Harrop, & MacMillan, 2015; Challender & Waterman, 2017; Heinrich et al., 2017). In particular, Chinese pangolin (*Manis pentadactyla*) populations are decreasing at an alarming rate due to habitat degradation and extensive illegal trade (Challender, Baillie, Waterman, & IUCN, 2012; Challender et al., 2015; Challender & Waterman, 2017; Chin & Pantel, 2009; Heinrich et al., 2017; Katuwal, Neupane, Adhikari, Sharma, & Thapa, 2015; Katuwal, Parajuli, & Sharma, 2016). Chinese pangolin is listed in the IUCN Red List as Critically Endangered (Challender et al., 2019) and on Appendix I by the Convention on International Trade in Endangered Species of Wild Fauna and Flora.

In Nepal, conservation action plan for Chinese pangolins suggest implementing management strategies to identify drivers of species occurrence and population dynamics (DNPWC & DoF, 2018). Further, the government of Nepal desires to understand the effectiveness of their protected areas in maintaining viable wildlife populations (DNPWC & DoF, 2018). A challenge to addressing these needs is that the occurrences of pangolin species in Nepal are not well documented due to their low abundance and nocturnal behavior (Bruce et al., 2017; Khwaja et al., 2019). Therefore, documenting Chinese pangolin occurrence through identification of sign, such as burrows (Katuwal, Sharma, & Parajuli, 2017; Thapa, Khatiwada, Nepal, & Paudel, 2014), could be beneficial for long‐term monitoring of pangolin species.

Because Chinese pangolin behavior limits the frequency of direct sightings, information on detection probabilities is a prerequisite for understanding their occurrence and habitat use. We employed an occupancy‐based modeling approach (MacKenzie, Nichols, Royle, Bailey, & Haines, 2006; MacKenzie, Nichols, Seamans, & Guitierrez, 2009; Miller et al., 2011; Royle & Dorazio, 2008) to estimate detection probability and occupancy of Chinese pangolins through repeated surveys of their active burrows. Due to government of Nepal's role on species conservation inside protected area (PA), we expected that site occupancy and detection probabilities estimates of Chinese pangolin would be greater in PAs than in non‐protected areas (non‐PA), in response to reduced human disturbance to habitat and greater food availability.

## 2 MATERIALS AND METHODS

### 2.1 Study area

**FIGURE 1:**
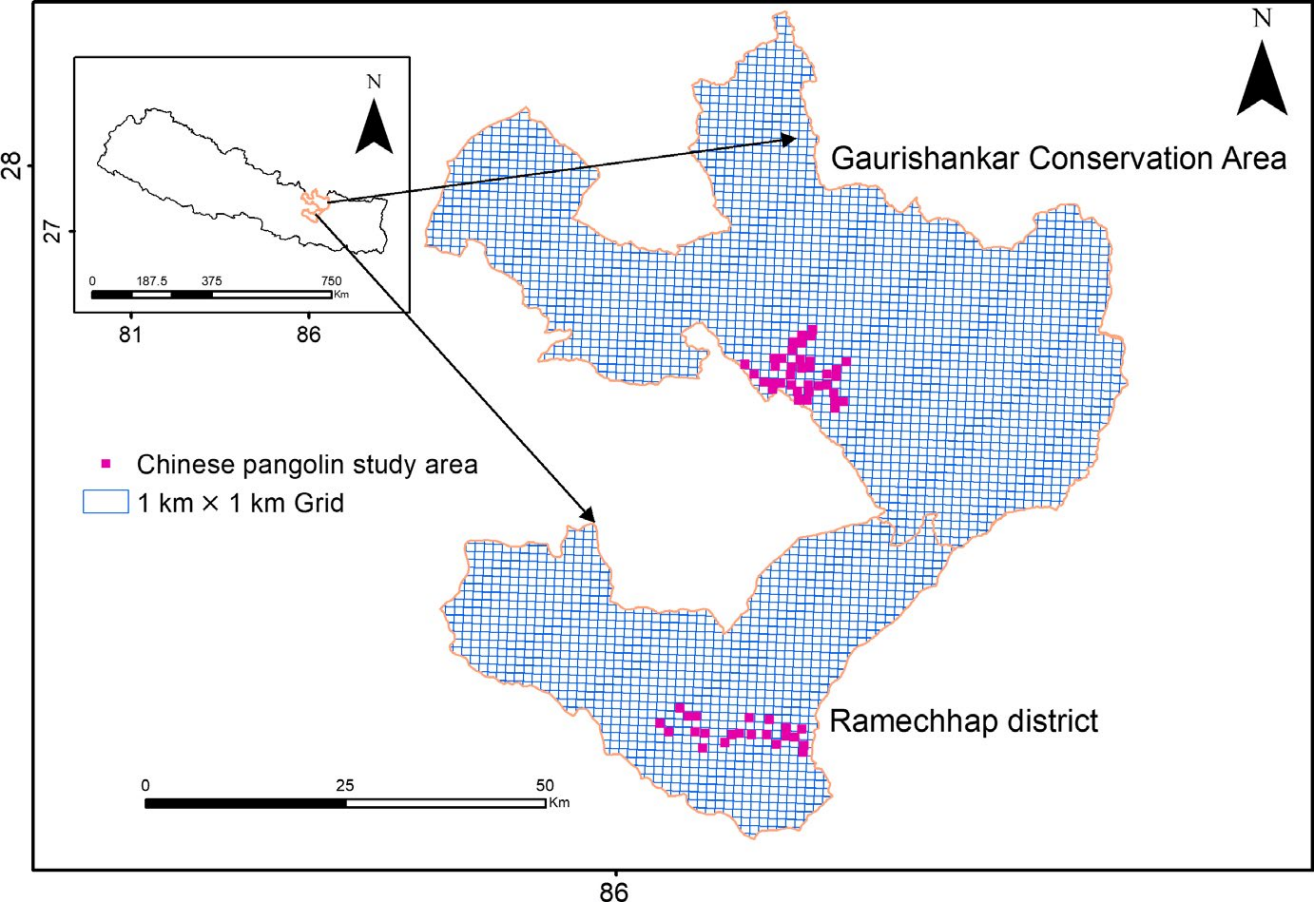
Chinese pangolin (*Manis pentadactyla*) study areas with 1 km^2^ grid in central Nepal

We conducted the study in a PA: Gaurishankar Conservation Area (GCA) (27°34′13.975″–28°10′22.065″N and 86°22′52.356″–86°11′5.257″E), and a non‐PA: Ramechhap District (27°49′55.04″–27°14′44.142″N and 86°9′0.474″–86°27′7.119″E), Nepal (Figure 1). The GCA was declared as a protected area on 19 July 2010 by the Government of Nepal (DNPWC, 2012). Gaurishankar Conservation Area falls within the Sacred Himalayan Landscape and comprises 2,179 km^2^, and ranged at elevations from 1,100 to 7,134 m above sea level. Nearly 12,000 households are within the GCA territory (DNPWC, 2012). The GCA is rich in floral diversity ranging from subtropical pine forests to alpine shrublands; common tree species include Chir pine *Pinus roxburghii*, Needlewood *Schima wallichina*, Nepalese alder *Alnus nepalensis*, Blue pine *Pinus wallichiana*, Patula pine *Pinus patula*, Woolly‐leaved oak *Quercus lanata*, Brown oak *Quercus semicarpifolia*, and Himalayan fir *Abies spectabilis*. Threatened mammal species in the GCA include Red panda *Ailurus fulgens*, Himalayan black bear *Ursus thibetanus,* and Snow leopard *Panthera uncia.* The area contains large numbers of fallen logs which support the occurrence of ants such as Big‐headed ant *Pheidole* spp., Saint valentine ant *Crematogaster* spp., and termites (SS Per. Obs).

Ramechhap District comprises 1,546 km^2^ and lies within the elevation of about 488 m–6,909 m above the sea level. There are 43,883 households within this district (CBS, 2011). The district has vegetation types ranging from subtropical forests to alpine shrublands; common tree species include Sweet orange *Citrus sinensis*, Common pear *Pyrus communis,* Nepali hog plum *Choerospondias axillaris*, Gros feuille *Litsea monopetala,* and Grey downy balsam tree *Garuga pinnata*. The district supports the occurrence of Barking deer *Muntiacus vaginalis*, Yellow‐throated Marten *Martes flavigula*, Common leopard *Panthera pardus,* Himalayan crestless porcupine *Hystrix brachyura,* Small Asian mongoose *Herpestes jervanicus,* Rhesus macaque *Macaca mulatta,* and Gray langur *Semnopithecus entellus* (NRCA, 2017), and some ants such as Big‐headed ant *Pheidole* spp., Saint valentine ant *Crematogaster* spp., and termites (SS Per. Obs). The Ramechhap District and GCA also harbor the threatened Chinese pangolin (DNPWC & DoF, 2018). Though a portion of Ramechhap District and the GCA overlap, we included the area of overlap as part of GCA as laws pertaining to protected areas were relevant to this area.

### 2.2 Chinese pangolin burrows survey

We conducted a preliminary survey during November 2018 to identify potential survey sites for Chinese pangolin (PA: Laduk, Suri, Bulun, Oran, Chankhu, and Khare; non‐PA: Saghutar, Deurali, Salu, Sunarpani, and Manthali). These study areas were selected based on accessibility and elevation constraints, that is, Chinese pangolins are not known to occur >2000 m elevation (DNPWC & DoF, 2018). We then established a 1 × 1 km grid within these districts and randomly selected 37 cells in the PA and 24 in the non‐PA for the survey based on the proportion of the study area. At the center of each cell, we established a 500 m line transect oriented to ensure water and food availability through scanning the surrounding. Along the transect, we established three 100 × 100 m plots separated by 100 m. We visited each plot for six consecutive days. On the first day, we recorded all burrows (active and inactive), and on the second through sixth days, we recorded new active burrows. We analyzed the data only of active burrows recorded after the first day made by Chinese pangolin. We followed DNPWC and DoF (2018) to distinguish the burrows made by pangolins with burrows made by other species. To identify burrows used by Chinese pangolin during each survey day, we used sticks with different colored ribbons. We marked old burrows (first day) using only a stick; on the second day, we marked the active burrows using a stick with white ribbon, and for the third, fourth, fifth, and sixth days, we used sticks with pink, purple, red, and yellow ribbon, respectively. We confirmed the burrow used by pangolin based on the fresh indirect evidences (pugmarks, scratches, and burrows).

We collected data on the active burrows (hereafter burrows) made by Chinese pangolin from 5 January to 23 February 2019 and 12 January to 9 March 2019 at the PA and non‐PA area, respectively. Altogether, we surveyed 183 (111 on PA and 72 on non‐PA) plots from both areas for six consecutive days. At each plot, we recorded 11 habitat covariates including distance to nearest water source (DW), elevation, slope, habitat type (forest or farmland), soil type based on color (red or brown), canopy cover (canopy) percentage, ground cover (ground) percentage, and presence and absence of food source (food) (ant nests, termite mounds). In addition, we also recorded four anthropogenic factors like distance to nearest human settlement (DS), distance to nearest road/foot trail (DR), distance to nearest livestock/sign (DL), and use of pesticides (pesticides). These covariates can influence the occurrence of Chinese pangolin (DNPWC & DoF, 2018; Katuwal et al., 2017; Wu, Liu, Ma, Xu, & Chen, 2003). We measured the distance of DS, DW, DL, and DR from the center of each plot by using a measuring tape, but if the distance was greater than 500 m from the center of each plot we measured the distance using a handheld global positioning system (GPS). We recorded the GPS location of the center of each plot and nearest DS, DW, DL, and DR, and the respective distances were estimated by overlaying these points in GoogleEarth. We recorded the slope at the center of each plot using a clinometer. We visually identified the habitat type, soil type, and pesticides as presence and absence and noticed by direct observation. The presence or absence of pesticides uses was identified after consultation with the respective land owners. At each plot, we also established five 10 m × 10 m subplots (four subplots at the corner and one at the center of each plot). From the center of each subplot, we measured the canopy cover and ground cover using 16 mega pixel an Android mobile app (canopy cover using Gap Light Analysis Mobile Application (GLAMA) and ground cover using Canopeo). We used the fisheye lens (present in GLAMA app) of radius 5.6 m to assess the tree Canopy Cover (CaCo) Index (Tichý, 2016), and simultaneously, we also measured the ground cover using the Canopeo app (Patrignani & Ochsner, 2015) from the height of two meters based on downward‐facing photographs. Both the apps are yet considered to be the powerful tools which captured the photographs and analyzed through the mobile phone application (Patrignani & Ochsner, 2015; Tichý, 2016). We averaged the percentage of canopy cover and ground cover from five different subplots and used the averaged data for the analysis. We noted the presence or absence of Chinese pangolin food sources (i.e., ant nests, termite mounds) in each plot. Distance to water source, distance to nearest human settlement, distance to nearest road, distance to nearest livestock/sign, canopy cover, ground cover, elevation, and slope were standardized before the analysis. We ran correlation analysis for variable selection and did not include variables with |r| > 0.7 in the same model (Dormann et al., 2012; Figure S1). We performed Moran’ I to test spatial autocorrelation of study plots.

### 2.3 Chinese pangolin occupancy and detection probabilities

In each study area, Chinese pangolin occupancy (Ψ) was estimated using a likelihood‐based method (MacKenzie, Nichols, Sutton, Kawanishi, & Bailey, 2005). Chinese pangolin burrow detection histories (H) were calculated for each plot (site) over five consecutive days. Thus, for each site and each occasion, “1” indicated the detection of burrow of Chinese pangolin and “0” indicated the nondetection of a Chinese pangolin burrow.

The probability of detecting Chinese pangolin burrow in five consecutive days given their occupancy at a given site was obtained from their detection history (PA: for site_1_ (H_5_) of 01011 would represent Chinese pangolin burrow detection on the second, fourth, and fifth days; non‐PA: for site_1_ (H_5_) of 01000 would represent Chinese pangolin burrow detection on the site two) and a detection probability of

(1) $$\begin{array}{c} \mathrm{Pr}\left(H_{1}=01011\right)=\Psi \left(1-P_{1}\right)P_{2}\left(1-P_{3}\right)P_{4}P_{5}\\ \;\;\;\;\;[\text{for protected area}] \end{array}$$

(2) $$\begin{array}{c} \mathrm{Pr}\left(H_{1}=01011\right)=\Psi \left(1-P_{1}\right)P_{2}\left(1-P_{3}\right)P_{4}P_{5}\\ \;\;\;\;\;[\text{for non-protected area}] \end{array}$$

Detection histories were produced for each of the two study areas and entered separately into unmarked packages in R (R Development Core Team, 2018). A logistic regression analysis was next performed to determine the covariates that best explain the Chinese pangolin burrow occupancy (Ψ) for each of the two study areas. We initially produced the simplest model, where occupancy and detection probability remained constant Ψ (.) p (.). We then constructed a global model containing all potential covariates for detection probability p (covariates) and allowed Ψ to vary by single covariates, Ψ (covariates). The potential covariates for detection probability were then allowed to vary, individually or in combination, while occupancy was either maintained with a single covariate or remained constant, that is, Ψ (covariates) p(covariates) and Ψ(.) p (covariates), respectively.

We used Akaike's information criterion adjusted for small sample sizes (AICc) to rank all the candidate models and calculate their Akaike weights (Burnham & Anderson, 2002). Models with ΔAICc of 0–2 of the best performing model provide most support (best models), while a ΔAICc of 4–7 has reduced support, and models with values >10 were considered not important (Burnham & Anderson, 2002). Once ΔAICc for each model was calculated, we selected the most parsimonious models using Akaike weights (W_i_), considering parsimonious models the top‐ranked models with when W_i_ >0.10 (Miller, 2014). We then summed the w_i_ of all the covariates (summed model weights ∑W_i_) across the candidate models to assess the importance of each covariate. We also compared Chinese pangolin occupancy and detection by averaging respective estimates along with standard error among the parsimonious models. The covariates with greater summed model weights were considered more important covariates in explaining heterogeneity in occupancy and detection (Andresen, Everatt, & Somers, 2014).

The most parsimonious models for the observed data were used to estimate the final Chinese pangolin active burrow occupancy (with standard errors [*SE*]), detection probabilities (with *SE*), and model precision on occupancy (*SE* [estimate]/PAO estimate × 100) (Linkie, Dinata, Nugroho, & Haidir, 2007) for the study area. The number of sites (s) that would be required to be surveyed (K) to achieve occupancy estimates with improved precision (i.e., *SE* = 0.05) was calculated by adopting a variance (Var) equation developed by MacKenzie et al. (2006), where

(3) $$Var\hat{\Psi }=\frac{\Psi }{s}\left[\left(1-\Psi \right)+\frac{{\left(1-p\right)}^{*}}{p^{*}-Kp{\left(1-p\right)}^{K-1}}\right]$$

if,

(4) $$p^{*}=\;1-{\left(1-p\right)}^{K}$$

is the probability of detecting the species at least once during K survey of an occupied site.

## 3 RESULTS

**FIGURE 2:**
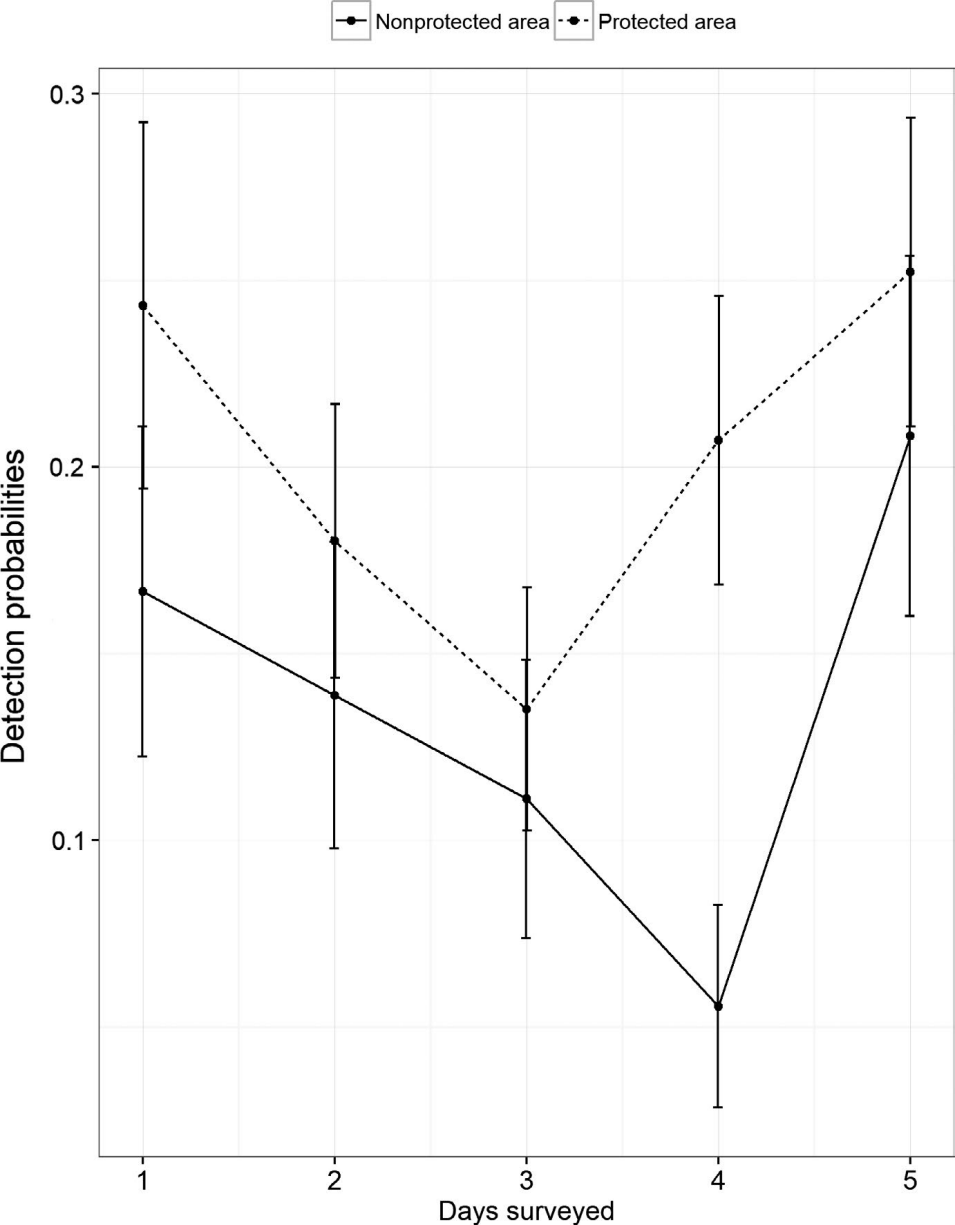
Detection of Chinese pangolin (*Manis pentadactyla*) during five survey days in a protected and non‐protected area of central Nepal

In the PA and non‐PA, the total number of Chinese pangolin burrows recorded was 138 and 105, respectively. The results of Moran's I indicated that sites within the PA and non‐PA were not spatially autocorrelation (Figure S2). The simplest model with constant occupancy and constant detection after pooling the data from PA and non‐PA was $\hat{\Psi }$ ± *SE*, 0.58 ± 0.05 (Model 1.6: Table 1, Figure 2). The potential differences among the candidate models, where $\hat{\Psi }$ and $\hat{p}$ were allowed to vary with different environmental covariates, found little support for the constant models, which had low AIC weightings (w_i_). The best and parsimonious models were Models 1.1–1.2. From the most parsimonious models, the Chinese pangolin occupancy was (Models 1.1–1.2) $\hat{\Psi }$ ± *SE*: 0.88 ± 0.11 and; $\hat{p}$ ± *SE* = 0.19 ± 0.05, respectively. Based on model $\hat{\Psi }$ (.) $\hat{p}$ (.), the detectability of Chinese pangolin burrow was 0.31 (*SE* = 0.026). As per the top‐ranked model, the Chinese pangolin burrow detectability was (see first two top Models) $\hat{p}$ = 0.19, *SE* = 0.05.

**TABLE 1:** Estimated Chinese pangolin (*Manis pentadactyla*) occupancy ( $\hat{\Psi }$ ) and detection probabilities ( $\hat{p}$ ) from top‐ranked models in Nepal

ID	Models	*N*	Δ AICc	W_i_	$\hat{\Psi }$ (1 ± *SE*)	$\hat{p}$ (1 ± *SE*)	Model precision
	covariates						
1.1	Ψ(Elevation)p( Forest+Slope+Ground+Red+Food+DR+DS+PA)	11	0.00	0.50	0.84 (0.09)	0.22 (0.05)	10.71
1.2	Ψ(Elevation)p(Farmland+Red+Food+DS+DL+DR+Canopy+PA)	11	0.17	0.46	0.92 (0.12)	0.16 (0.04)	11.08
1.3	Ψ(.)p(Farmland+Red+Food+DR+DS+PA)	8	7.28	0.01	0.76 (0.08)	0.31 (0.05)	10.52
1.4	Ψ(Elevation)p(DW+non‐PA)	5	7.75	0.01	0.70 (0.07)	0.35 (0.03)	10.00
1.5	Ψ(Elevation)p( Forest+Brown+Food+DL+DR+DS+non‐PA)	10	8.06	0.00	0.81 (0.09)	0.27 (0.06)	11.11
1.6	Ψ(.)p(.)	2	13.33	0.00	0.58 (0.05)	0.31 (0.026)	8.62
1.7	Model averaged				0.77 (0.08)	0.27 (0.05)	10.34

Note

The covariates used in the study were habitat types (forest or farmland), soil type (red or brown), tree canopy, ground cover, distance to nearest human settlement (DS), distance to nearest road/foot trail (DR), distance to nearest livestock/sign (DL), food source, elevation, and slope after pooling the data from a protected (PA) and non‐protected (non‐PA) areas in Nepal. Ψ is the probability a site is occupied by Chinese pangolin, and p is the probability of detecting Chinese pangolin in the jth survey where Ψ (.)p(.) assumes that pangolin presence and detection probability are constant across sites, $\hat{\Psi }$ is the estimated over all occupancy probability, K is the number of parameters in the model, ΔAICc is the difference in AIC values between each model with the lowest AIC model, and W_i_ is the AIC model weight.

**FIGURE 3:**
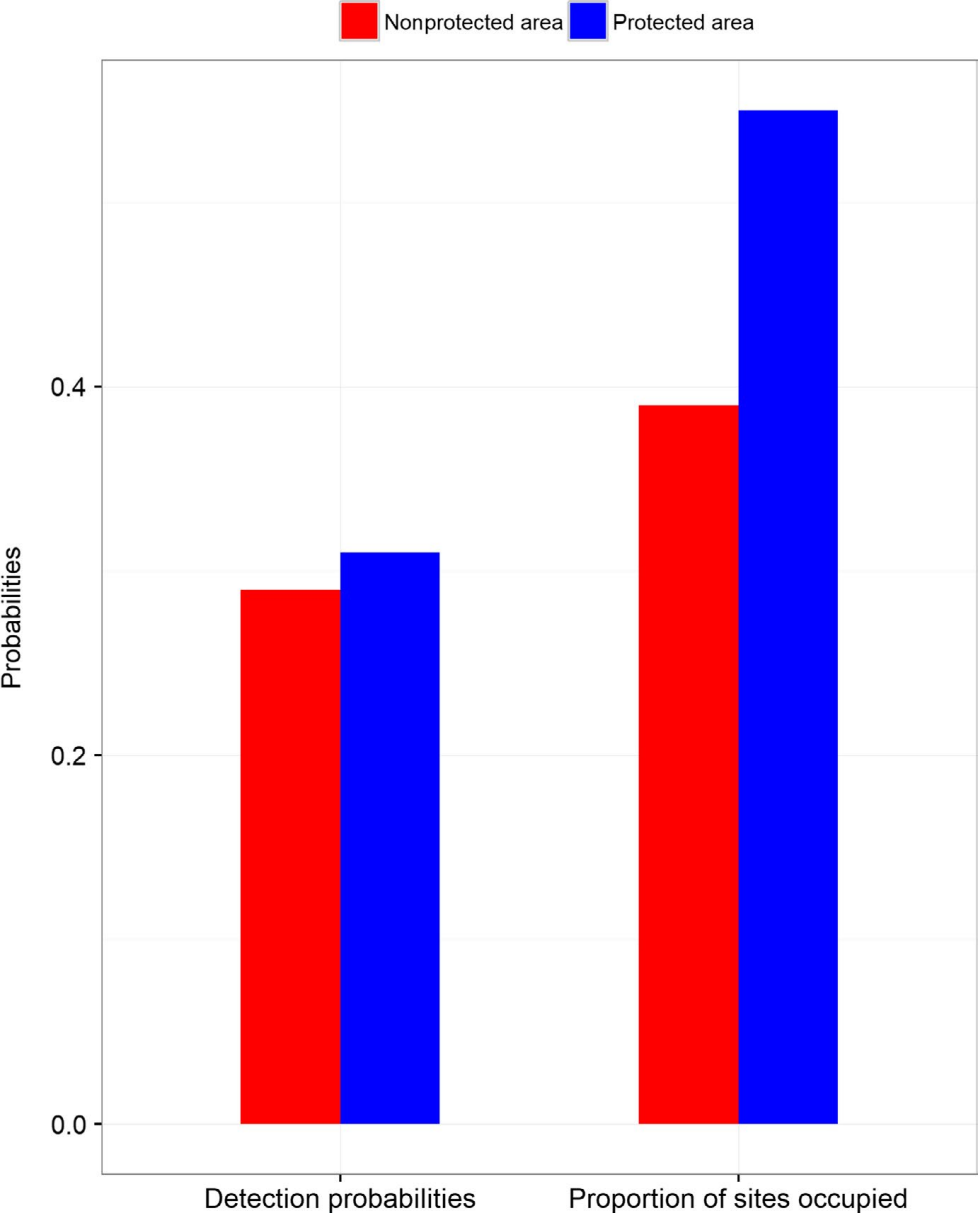
Detection probabilities and proportion of sites occupied by Chinese pangolin (*Manis pentadactyla*) in a protected and non‐protected area of central Nepal

**FIGURE 4:**
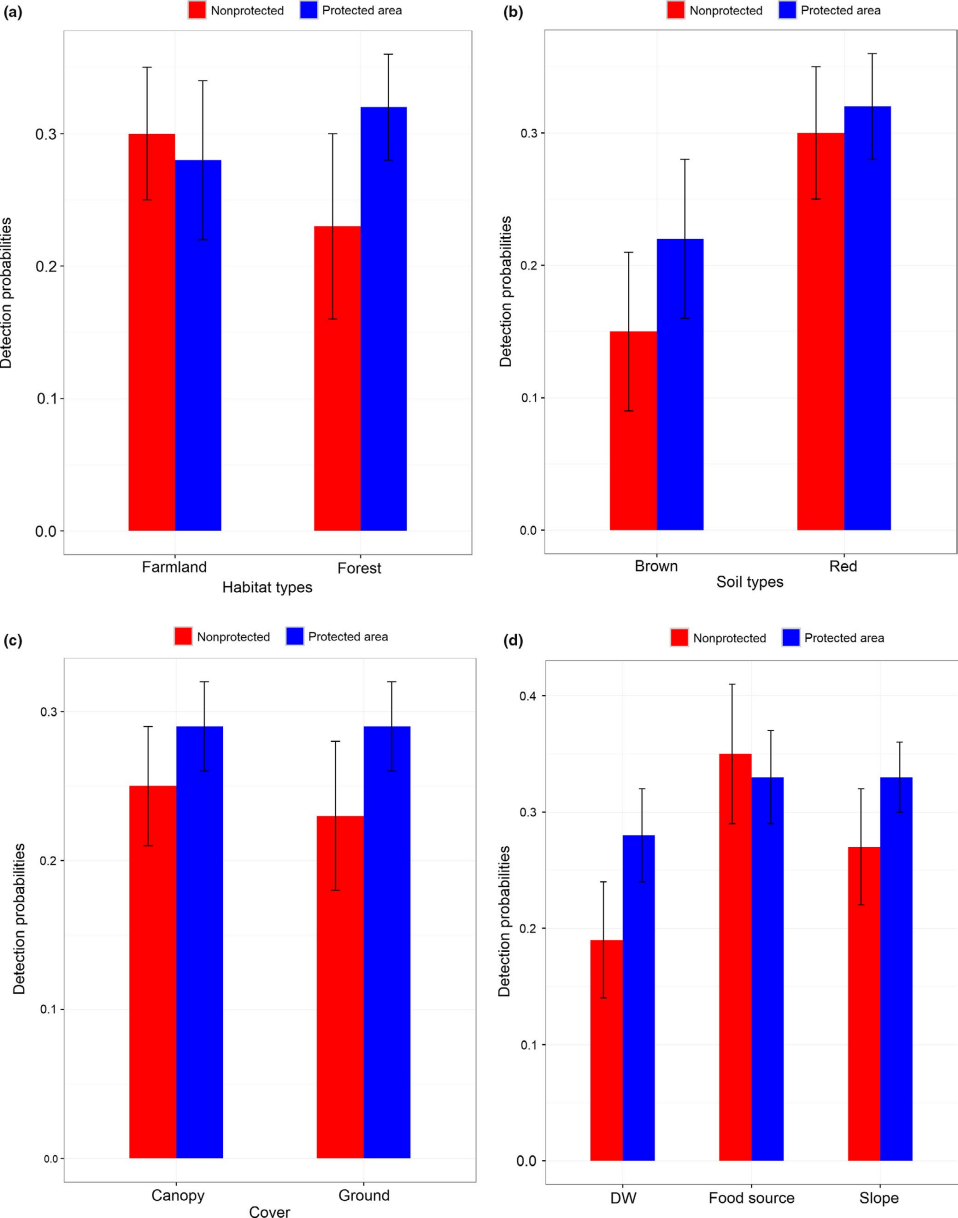
(a) Detection probabilities of Chinese pangolin (*Manis pentadactyla*) by habitat type in a protected and non‐protected area; (b) detection probabilities of Chinese pangolin with soil types in Protected and non‐protected area; (c) detection probabilities of Chinese pangolin with cover in Protected and non‐protected area; and (d) detection probabilities of Chinese pangolin with DW, food source, and slope in protected and non‐protected area

All models had precision <30% (Table 1). No single model emerged as the top‐ranked model, that is, w_i_ >0.90 so the model averaged occupancy value was taken as the final estimate (Model 1.7, Table 1). Furthermore, the average Chinese pangolin burrow occupancy and detection probabilities were $\hat{\Psi }$ ± *SE* = 0.77 ± 0.08; $\hat{p}$ ± *SE* = 0.27 ± 0.05, respectively. The naïve occupancy estimate (observed proportion of sites occupied) for Chinese pangolin was 0.49 over the five sampling occasions (Figure 3). The detection probabilities of Chinese pangolin were greater in PA ( $\hat{p}$ ± *SE* = 0.33 ± 0.03) than compared to non‐PA ( $\hat{p}$ ± *SE* = 0.25 ± 0.04) (Table 2; Figure 2). The ∑ W_i_ for major factors with respect to detection on pooling both the areas were as follows: red soil (97%), food source (97.6%), DR (97.9%), PA (97%), and DS (97.9%) and with respect to occupancy was elevation (97.9%) (Table 3). Among these greatest ∑ W_i_, elevation (coefficient ± *SE*: 0.37 ± 0.28), red soil (coefficient ± *SE*: 0.71 ± 0.39), food source (coefficient ± *SE*: 0.99 ± 0.52), and PA (coefficient ± *SE*: 1.23 ± 0.59) were positively associated with Chinese pangolin burrows, while DR (coefficient ± *SE*: −0.62 ± 0.33) and DS (coefficient ± *SE*: −0.55 ± 0.24) were negatively associated, and the detection probabilities were differ in PA and n‐PA according to the sources (Figure 4a‐d). Pooled detection probabilities of Chinese pangolin burrows were greater in the PA (0.33 ± 0.03) and positively influenced by food source (0.34 ± 0.03), red soil (0.32 ± 0.03), and DS (0.30 ± 0.03) (Table 2). Finally, to achieve a model precision for the combined study area with *SE* = 0.05 (Equations 3 and 4), based on data collected over five sampling occasions, we estimated 250 site surveys would be required when pooling both the plots of survey areas.

**TABLE 2:** Detection probabilities of Chinese pangolin (*Manis pentadactyla*) burrow by habitat types, soil type, cover, distance to nearest human settlement (DS), distance to nearest road (DR), distance to nearest livestock/sign (DL), food source, and slope after pooling the data from a protected (PA) and non‐protected (non‐PA) areas in Nepal

Covariates		Detection probabilities ± *SE*
Habitat types	Forest	0.31 ± 0.03
	Farmland	0.29 ± 0.04
Soil types	Red	0.32 ± 0.03
	Brown	0.21 ± 0.005
Cover	Canopy	0.28 ± 0.03
	Ground	0.28 ± 0.03
Areas	PA	0.33 ± 0.03
	non‐PA	0.25 ± 0.04
DW		0.25 ± 0.03
Food source		0.34 ± 0.03
Slope		0.31 ± 0.03
DL		0.29 ± 0.03
DR		0.29 ± 0.03
DS		0.30 ± 0.03
Pesticides		0.12 ± 0.09

**TABLE 3:** Estimate, standard error, confidence interval, and ∑ W_i_ of covariates in both the PA and non‐PA

Covariates	Coefficient ± SE	z	Upper CL	Lower CL	∑ W_i_ (%)

Elevation	0.37 ± 0.28	1.36	−0.94	0.41	97.9
Slope	0.04 ± 0.23	0.17	0.41	0.49	50
Canopy	−0.56 ± 0.27	−2.05	−1.10	0.04	46
Ground	1.77 ± 0.47	3.76	0.92	2.78	50
Red	0.71 ± 0.39	2.47	−0.28	2.71	97
Brown	−1.12 ± 0.21	2.14	−0.28	2.14	1
Forest	0.87 ± 0.53	1.64	−0.16	1.94	50.9
Farmland	−0.87 ± 0.53	−1.64	−1.94	0.16	47
DW	0.57 ± 0.24	2.40	0.11	1.05	1
Food	0.99 ± 0.52	1.92	0.02	2.03	97.6
DS	−0.58 ± 0.24	−2.37	−1.09	−0.14	97.9
DR	−0.62 ± 0.33	−1.88	−1.64	1.31	97.9
DL	−1.83 ± 0.39	−4.61	−0.11	2.67	46.9
PA	1.23 ± 0.59	2.10	0.12	2.45	97
Non‐PA	−1.23 ± 0.59	2.10	−0.25	−0.12	1.9

Note

The covariates used in the study were habitat types (forest or farmland), soil type (red or brown), tree canopy, ground cover, distance to nearest human settlement (DS), distance to nearest road/foot trail (DR), distance to nearest livestock/sign (DL), food source elevation, and slope after pooling the data from a protected (PA) and non‐protected (non‐PA) areas in Nepal.

## 4 DISCUSSION

Globally, the population of Chinese pangolin is declining rapidly, and therefore, monitoring the occurrence and habitat associations of this species are crucial. To our knowledge, we provide the first occupancy modeling based on habitat use of Chinese pangolin.

The Chinese pangolin burrow detectability was greater in the PA than in the non‐PA. We suggest this is a consequence of reduced human disturbance through management intervention for wildlife conservation in GCA (PA). Healthy forests support the occurrence of Chinese pangolin (Katuwal et al., 2017; Sharma et al., 2020), which more common in PAs than non‐PAs in Nepal, and likely supports higher occurrence of prey species. Chinese pangolin's occurrence was greater in PAs reduced disturbances, typically >1,000 m from the human settlements, livestock grazing, and road access (Katuwal et al., 2017; Wu et al., 2003). However, 51% of plots with burrows were nearer to human settlements (<1,000 m). Many settlements practicing agriculture are sparsely distributed within forested areas. Both forest and agricultural lands support the occurrence of Chinese pangolin (Katuwal et al., 2017; Sharma et al., 2020) and though livestock may not directly disturb Chinese pangolins, livestock guard dogs, and people do pose threats. We suggest the observed response of greater Chinese pangolin occupancy nearer to human settlements is in part a consequence of the dispersion of human settlements within forested areas. The GCA is mainly targeted for the conservation of threatened species, such as Himalayan black bear *Ursus thibetanus,* but Chinese pangolin may have cobenefitted from such effort. GCA regularly implements participation and awareness programs for local people (NTNC, 2015) which have been demonstrated to benefit species conservation (Bajracharya, Gurung, & Basnet, 2007). Generally, wildlife reserve establishment with efficient management practices can lead to the conservation of species threatened with extinction (Hoffmann et al., 2010). For example, average occupancy of dhole was found high within the reserve of Western Ghats of India (Srivastha, Karanth, Kumar, & Oli, 2019).

Chinese pangolin burrow use was not always detected when present, as the detection probabilities ( $\hat{p})$ in each study area were less than 1.0. The naïve occupancy estimate, which assumes *p* = 1.0, was found to underestimate the occupancy value of $\hat{\Psi }$ (.) $\hat{p}$ (.) by 12.00%–14.00% across the two study areas and demonstrates the need to incorporate detection probability to produce more reliable occupancy estimates. Detection probability can be affected by many factors including local density and weather for amphibians (Bailey, Simons, & Pollock, 2004; Pellet & Schmidt, 2005) or road proximity for sun bears (Linkie et al., 2007).

Chinese pangolin burrows were expected to differ within the study areas because of variation in environmental covariates at each plot. The Chinese pangolin's burrow detection is determined by forest, red soil, and food source in PA while food source along with the farmland in non‐PA. Elevation was the important contributing factors for estimating occupancy in non‐PA compared to PA because Chinese pangolin were assessed from low to high elevations (about 600–1400 m). This finding corroborates an earlier study by DNPWC and DoF (2018) that recorded burrows at 500–2000 m elevation. Generally, Chinese pangolins prefer slopes <50° (Wu et al., 2003), and in our study, most burrows occurred on 15–22° slopes in the PA. This occurrence is probably due to less disturbances and more abundant fallen logs on these slopes which are important for ants and termites. Though fallen log collection in PAs is prohibited, collection that does occur is generally on less steep slopes. Higher livestock grazing in the non‐PA can reduce the moisture content of understory vegetation leading to reduced habitat suitability for detritivores (Bromham, Cardillo, Bennett, & Elgar, 1999), which in turn reduces the prey base of Chinese pangolin. Use of pesticides was also reported from non‐PA which might reduce the prey base of Chinese pangolin occurrence. We suggest occurrence of Chinese pangolin burrows in red soil habitats was a consequence of increased availability of food compared to brown soil (SS, Pers. Obs.). Further, the PA had less human disturbance than in non‐PA which could result in greater food availability in the PA. However, Chinese pangolin have been detected in farmlands in non‐PAs, including near human disturbance (Katuwal et al., 2017).

From our combined study areas (183 plots), we predicted that a total of 250 plots would need to be surveyed to obtain $\hat{\Psi }$ estimates with 0.05_s_. This could be achieved by increasing the number of plots within each study area and reducing the number of search occasions (e.g., 3) per plot. Overall, $\hat{\Psi }$ (.) models received greater support in the model selection procedure, as indicated by higher AIC weightings.

As Chinese pangolins are nocturnal and elusive (DNPWC & DoF, 2018), occupancy estimates of pangolins based on direct observation are difficult (Willcox et al., 2019). We found that using an indirect sign survey was suitable for estimating the occupancy of Chinese pangolin to address the problem of inadequate direct sightings. To validating the indirect sign and survey especially for the study of mammals, occupancy models are considered a powerful approach (Yarnell et al., 2014). However, our survey did not meet the assumption of abundance models of occupancy (Conroy, Runge, Barjer, & Schofield, 2008; Nichols, Hines, MacKenzie, Seamans, & Guiterrez, 2007; Royle & Nichols, 2003). We recommend further applications of occupancy modeling using alternative data types (e.g., remote cameras) to improve reliability of estimates of Chinese pangolin, particularly detectability. In addition, refined data and associated models would facilitate the identification of spatially explicit priority areas to improve conservation of the Critically Endangered Chinese pangolin in both PAs and non‐PAs.

## CONFLICT OF INTEREST

None.

## AUTHORS CONTRIBUTIONS

SS, HPS, and HBK designed the study. SS and CC carried out the field survey. SS, HPS, and HBK did data analysis. SS, HPS, CC, HBK, and JLB wrote and finalized the manuscript.

## Supporting information

Fig S1‐S2

## Data Availability

All the relevant data used in this study will be archived in Dryad after the acceptance of the manuscript. https://doi.org/10.5061/dryad.tqjq2bvvd
