# Supplementary material for: Estimating occupancy of Chinese pangolin (Manis pentadactyla) in a protected and non‐protected area of Nepal
Source: Ecol Evol. 2020 Mar 17;10(10):4303–13. doi: 10.1002/ece3.6198 (PMC7246206; doi:10.1002/ece3.6198)
Supplement: Supplementary file 1 — Fig S1‐S2 [file ECE3-10-4303-s001.docx]

**Supplementary Figures**

**
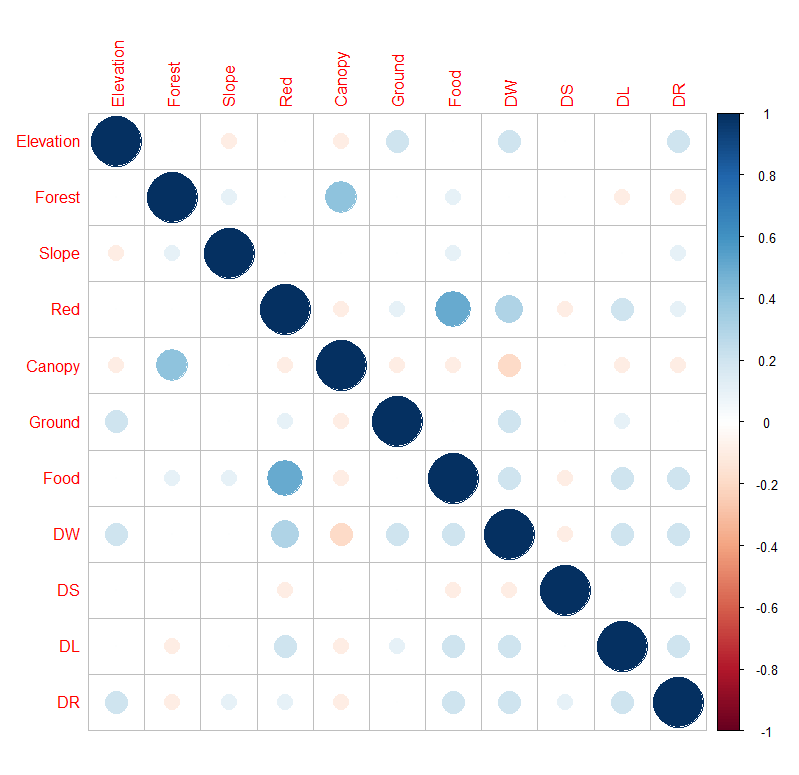
**

Figure S1. Correlation between variables elevation, slope, forest, soil type based on color (red or brown), canopy percentage, ground cover percentage, food source (presence/absence), distance to nearest water source (DW), distance to nearest human settlement (DS), distance to nearest road/foot trail (DR), distance to nearest livestock/sign (DL) and use of pesticides (Yes/No). The continuous variables were standardized before the analysis.


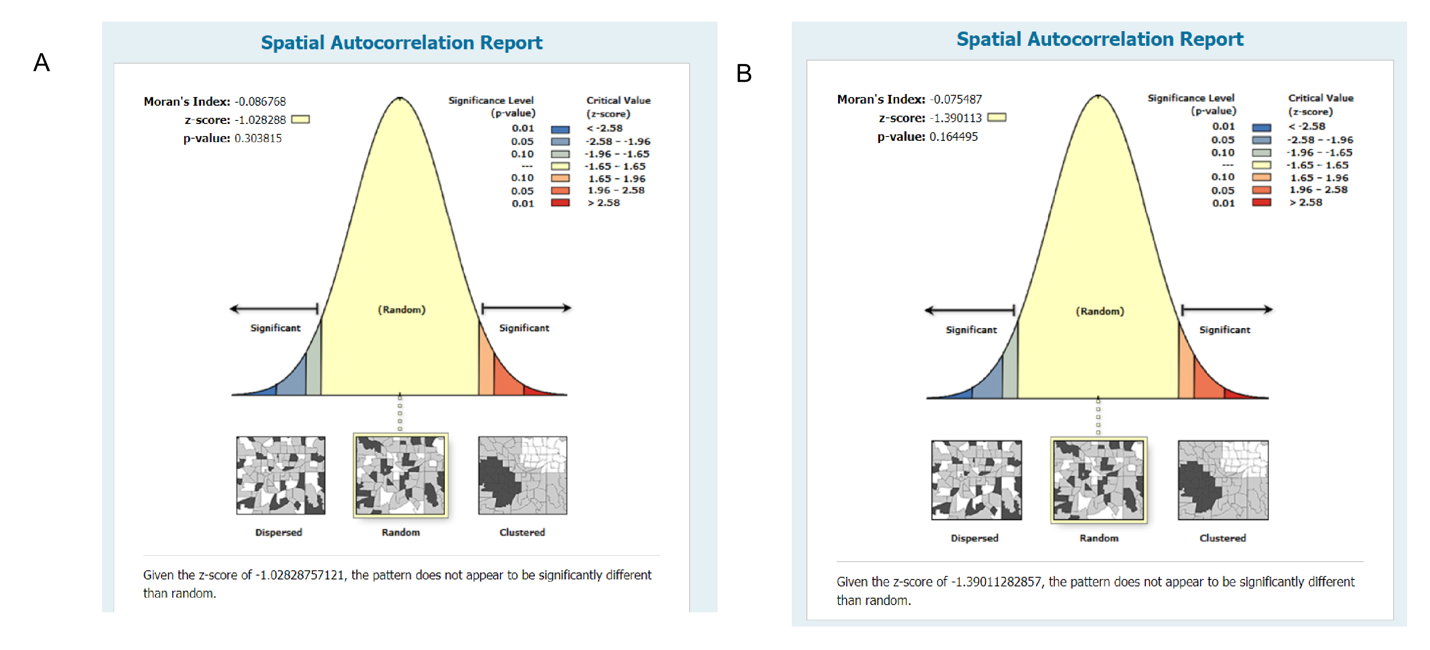


Figure S2. Moran’ I test to know whether the data were in spatial autocorrelation in Protected area (A) and non-protected area (B).
